# Bending Vibration Characteristics of a Novel Piezoelectric Composite Trilaminar Vibrator

**DOI:** 10.3390/ma14133661

**Published:** 2021-06-30

**Authors:** Ning Lv, Chao Zhong, Jiacheng Wang, Likun Wang

**Affiliations:** Beijing Key Laboratory for Sensor, Beijing Information Science & Technology University, Beijing 100192, China; 20192289@bistu.edu.cn (C.Z.); 2019020178@bistu.edu.cn (J.W.)

**Keywords:** resonance frequency, bending vibration, vibration displacement, finite element simulation

## Abstract

In this work, the bending vibration characteristics of the 2-2 piezoelectric composite trilaminar vibrator are studied by the finite element simulation and experiment. The simulation results show that the trilaminar vibrator has lower resonant frequency and larger vibration displacement under the fixed boundary condition compared with that of the free boundary condition, and its performance is relatively good. Then, the 2-2 piezoelectric composite and piezoelectric ceramic trilaminar vibrators are fabricated and their performances are tested under the fixed boundary condition. The experimental and simulation results show that the vibrator has pure bending vibration characteristics in the frequency band of 1.2–1.4 kHz, especially the 2-2 piezoelectric composite vibrator, which has lower frequency, higher electromechanical coupling coefficient and larger bending vibration displacement; thus, the 2-2 piezoelectric composite trilaminar vibrator is a better choice for the fabrication of a low-frequency transducer.

## 1. Introduction

As an important part of the global life system, the ocean is a precious wealth which calls for human society to achieve sustainable development [1]. Coastal countries have focused on the development and utilization of marine resources with the development of science and technology [2]. The underwater acoustic transducer, an important component of ocean detection, has played an important role in underwater detecting, classifying, locating and tracking underwater targets [3,4]. The performance of underwater acoustic transducers is required to be higher with the rapid development of ocean exploration technology which makes the transducer develop in the direction of low frequency and high power as well as small size and broadband [5]. The bending laminated transducer is a common low-frequency transducer which uses the bending vibration of piezoelectric ceramics to radiate energy and has the advantages of simple structure, small size and light weight [6]. Such a transducer is an ideal low-frequency vibration sound source and can produce much lower resonance frequency than longitudinal, thickness and radial vibration frequency under the same geometric size [7]. At present, the laminated transducers are mainly divided into two structures: bilaminar and trilaminar transducers. The trilaminar transducer has the advantages of low resonance frequency, simple structure and stable performance compared with the bilaminar transducer [8]. It is widely used in underwater acoustic technology, marine development, acoustic logging and other research fields. Its working mechanism and acoustic characteristics have attracted the attention of relevant researchers.

In recent years, many reports on trilaminar bending vibration transducers have been produced. Zheng et al. [9] carried out the finite element design of trilaminar dipole acoustic logging transducer and systematically discussed the influence of the transducer size and structure parameters on the frequency characteristics and radiation characteristics of the transducer. Sun [10] studied the relationship between the performance and size of the transducer and deduced the basic vibration equation of isotropic homogeneous thin plate with the knowledge of elastic mechanics based on the assumption of thin plate theory. Li et al. [11] simulated and tested the trilaminar transducer and the results showed that the medium and mechanical boundary conditions have influence on the vibration mode and resonant frequency of the transducer. Ling et al. [12] used the Rayleigh method to study the vibration characteristics of trilaminar disk flexural vibration transducer under simply supported conditions and numerically analyzed the variation of resonant frequency and effective electromechanical coupling coefficient with the size parameters of the transducer. Zhang [13] studied the bending vibration of the trilaminar curved disk transducer and theoretically calculated the effects of boundary conditions and structural parameters on the resonant frequency and effective electromechanical coupling coefficient of the transducer. Liu et al. [14,15] simulated and studied the trilaminar ring and dodecahedral transducer which provided theoretical support for the subsequent development of this type of transducer. Xu et al. [16] designed a double-sided trilaminar curved hydrophone which realized high sensitivity receiving of low-frequency sound waves.

The above research status indicates that all the reported trilaminar transducers are made of piezoelectric ceramics. Although piezoelectric ceramics have the advantages of high electromechanical coupling coefficient, wide range of dielectric constant, low loss, convenient fabrication and low price, they also have the disadvantages of narrow frequency band, high Q value and poor sensitivity. Therefore, in recent years, many researchers use piezoelectric composites instead of piezoelectric ceramics, hoping to make up for the shortcomings of piezoelectric ceramics. Jin [17] studied the structure and properties of piezoelectric materials, and the results showed that piezoelectric composites have the advantages of both piezoelectric ceramics and polymeric materials. Compared with traditional piezoelectric ceramics (or piezoelectric single crystal), it has better flexibility and machining performance and overcomes the shortcomings of fragile and difficult to process. In addition, it has low density *ρ*, low sound velocity *v*, and is easy to achieve acoustic impedance matching with air, water and biological tissues. Chen et al. [18] made a comparative analysis of the performance of piezoelectric composite and piezoelectric ceramic transducers, and the results showed that the piezoelectric composites have a purer vibration mode, better broadband transceiver performance and higher sensitivity compared with ordinary PZT piezoelectric ceramics. Liu et al. [19] made a comparative analysis on the performance of 2-2 piezoelectric composite bilaminar vibrator and piezoelectric ceramic bilaminar vibrator, and the results showed that the resonant frequency of 2-2 piezoelectric composite materials is lower, and the electromechanical coupling coefficient and vibration displacement are larger compared with piezoelectric ceramic materials. Chen et al. [20] designed a novel broadband radial vibration ultrasonic transducer which was made of 2-2 piezoelectric composite materials, and the results show that the novel composite material radial transducer has larger emission voltage response amplitude, nearly doubled working bandwidth and better acoustic matching compared with the traditional piezoelectric ceramic radial transducer.

In conclusion, the piezoelectric composites have the advantages of single vibration mode, broadband, high electromechanical coupling coefficient, and good transmission voltage response and receiving voltage sensitivity compared with piezoelectric ceramics, which can compensate the shortcomings of piezoelectric ceramics. Therefore, in this work, the piezoelectric composite material is used to replace the traditional piezoelectric ceramic material, and a novel rectangular piezoelectric composite trilaminar vibrator is designed and prepared. Its frequency is reduced, and its vibration displacement and bandwidth are improved compared with the current transducers, which lays a foundation for the preparation of a high-performance low-frequency transducer.

## 2. Structure of the Piezoelectric Composite Trilaminar Vibrator

**Figure 1:**
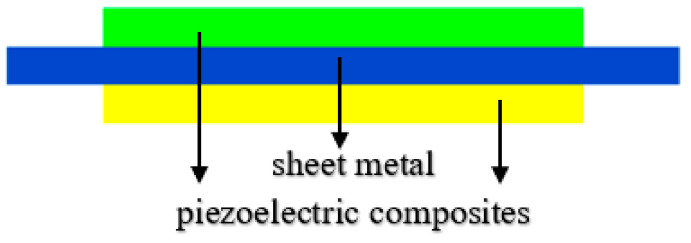
The basic structure of the trilaminar vibrator.

**Figure 2:**
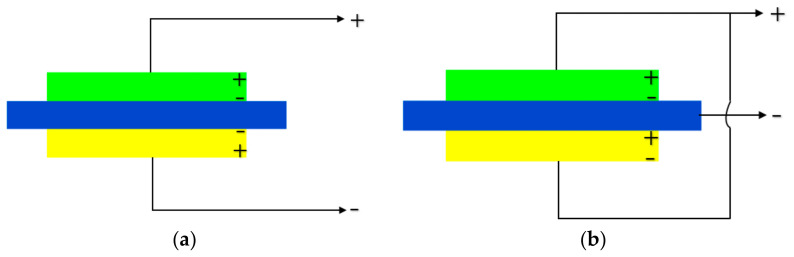
Two kinds of circuit connection modes: (**a**) series mode; (**b**) parallel mode.

The structure of the trilaminar vibrator is very simple. A piece of metal can be glued between the bilaminar vibrator sheets to form the trilaminar vibrator, which is convenient for supporting and circuit connection. Figure 1 shows the basic structure of the trilaminar vibrator, in which the green and yellow parts represent the piezoelectric composite materials, and the blue part denotes the metal sheet. At present, there are mainly two kinds of circuit connection modes of the trilaminar vibrator, among which the series connection mode is shown in Figure 2a and the parallel connection mode is shown in Figure 2b. When the polarization direction of the upper and lower piezoelectric sheets is opposite, the power supply is connected in series. When the polarization direction of the upper and lower piezoelectric sheets is the same, the power supply is connected in parallel. The composite trilaminar vibrator is developed with this method because the method shown in Figure 2a is relatively simple.

**Figure 3:**
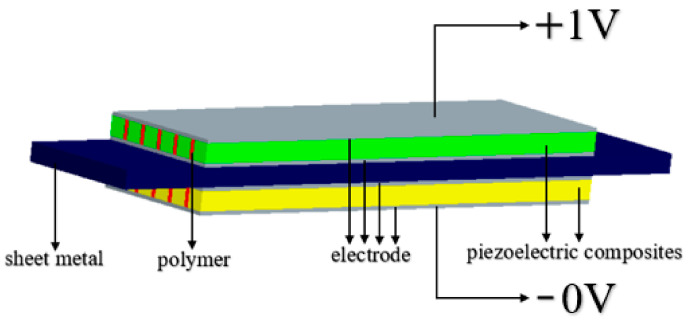
2-2 piezoelectric composite trilaminar vibrator.

Figure 3 shows the final piezoelectric composite trilaminar vibrator structure, one of which is composed of 2D connected piezoelectric ceramic thin plates periodically arranged in the 2D connected polymer and connected in a 2-2 way. The piezoelectric ceramic adopts PZT-5A, as shown in the yellow and green parts of the figure; the polymer adopts silicone rubber, as shown in the red part of the figure; and the metal sheet adopts aluminum in the middle, as shown in the blue part of the picture. The thickness of the metal sheet is set as 1/3 of the total thickness of the vibrator because the electromechanical coupling coefficient of the vibrator is the highest when the thickness of the three laminates in the trilaminar vibrator is equal [21]. The 2-2 piezoelectric composites have the advantages of low impedance, low mechanical quality factor and wide frequency band compared with traditional piezoelectric ceramics. The structure vibrates along the length direction, and the piezoelectric ceramic column of 2-2 structure can support the vibrator. The vibrator adopts the principle of Figure 2a for electrode connection, in which the grey part represents the electrode of the vibrator, the upper and lower electrodes are both positive, and the part bonded to the metal sheet in the middle is negative. The vibrator can form bending vibration under the driving of voltage because the polarization direction of the upper and lower laminations is opposite, and the voltage shown in the figure is added.

## 3. Finite Element Simulation of the Trilaminar Vibrator

Finite element simulation software ANSYS is used to simulate the trilaminar vibrator to design a piezoelectric vibrator with excellent performance [22,23,24]. The influences of the material types and boundary conditions of the vibrator on the resonant frequency, electromechanical coupling coefficient and vibration displacement are calculated.

Firstly, the quasi-physical model is established according to the structure of the trilaminar vibrator. The length of the piezoelectric vibrator is 50 mm, the width is 14.8 mm, the thickness is 2 mm, and the volume fraction is 78.12%. Then, the material parameters of the piezoelectric phase, the polymer phase and the metal sheet are defined. The solid5 is selected as the unit type of the piezoelectric phase and the solid45 is selected as the unit type of the polymer phase and the metal sheet. The piezoelectric phase is PZT-5A, the polymer phase is silicone rubber and the metal sheet is aluminum. The parameters are shown in Table 1.

**Figure 4:**
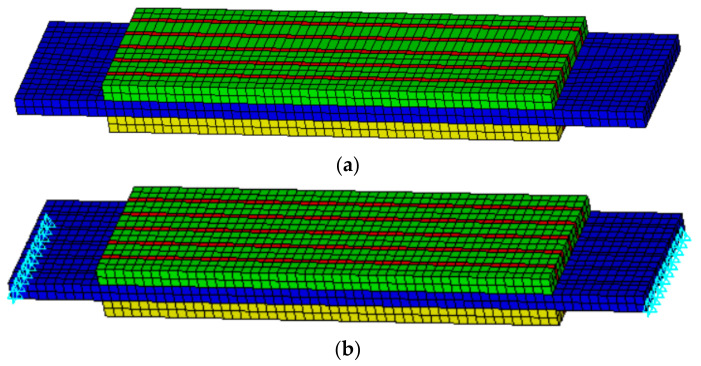
Finite element model with meshing: (**a**) free boundary; (**b**) fixed boundary.

The hexahedral element is used to generate the finite element mesh, and the height of each row of FEs is 1 mm. The finite element model after meshing is shown in Figure 4, in which green represents the forward polarized piezoelectric phase, yellow shows the reverse polarized piezoelectric phase, red denotes the polymer phase, and blue the indicates metal sheet. Figure 4a shows the finite element model under the free boundary condition which needs to keep the left and right sides free. Figure 4b shows the finite element model under the fixed boundary condition which needs to fix the thickness direction of the left and right sides of the middle metal sheet.

**Figure 5:**
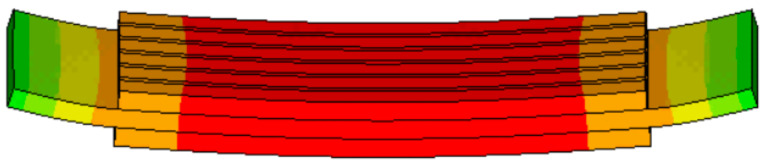
The vibration mode of the trilaminar vibrator.

Quantities of 0 and +1 V alternating voltage are applied to the upper and lower surfaces of the established finite element model, and the Harmonic option in ANSYS (15.0, ANSYS, Inc., Pittsburgh, PA, USA) is selected as the analysis type. The frequency range is set as 1–9 kHz, the scanning substep is set as 200 and the damping coefficient is set as 0.02; then, calculation is performed. After the calculation, the admittance frequency curve is read with an ANSYS post-processor and the vibration mode at the resonance frequency point is observed. The vibration mode of the trilaminar vibrator is shown in Figure 5, which is the bending vibration in the transverse direction.

**Figure 6:**
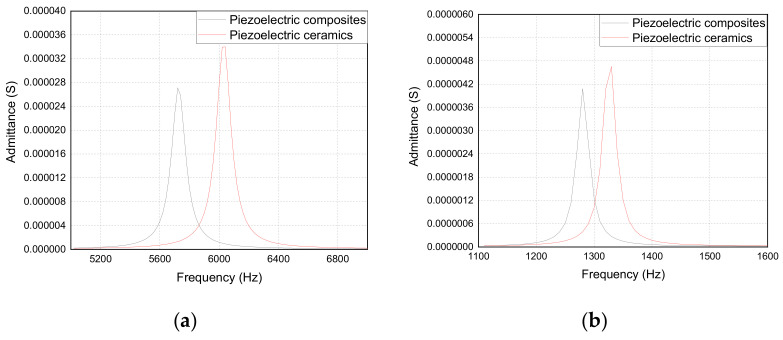
Admittance curve of the trilaminar vibrator: (**a**) free boundary; (**b**) fixed boundary.

Figure 6 shows the simulation results of the admittance curve of the trilaminar vibrator. Figure 6a demonstrates that the 2-2 piezoelectric composite vibrator has a lower resonant frequency than the piezoelectric ceramic vibrator. Figure 6b demonstrates that the fixed boundary condition can greatly reduce the resonant frequency of the trilaminar vibrator, which can make the resonant frequency become a quarter of that of the free boundary condition.

**Figure 7:**
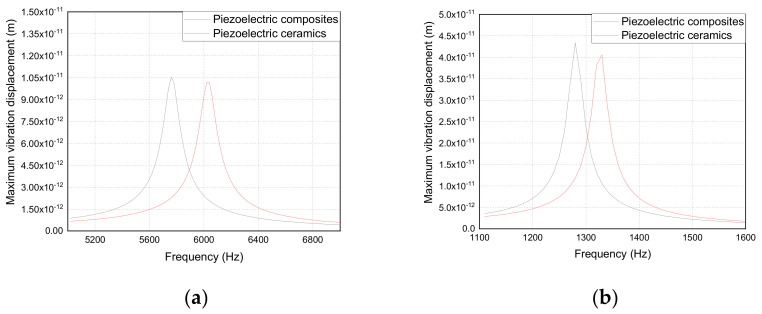
The vibration displacement curve of the trilaminar vibrator: (**a**) free boundary; (**b**) fixed boundary.

Figure 7 shows the simulation results of the vibration displacement of the trilaminar vibrator. Figure 7a demonstrates that the 2-2 piezoelectric composite vibrator has a larger vibration displacement than the piezoelectric ceramic vibrator. Figure 7b demonstrates that the vibration displacement of the vibrator can be greatly increased and is approximately three times that of the free boundary under the fixed boundary condition.

In summary, the trilaminar vibrator has lower resonance frequency and larger vibration displacement under the fixed boundary condition compared with that of the free boundary condition. Therefore, the fixed boundary is used to study the bending vibration characteristics of the trilaminar vibrator.

## 4. Preparation and Performance Test of the Trilaminar Vibrator

The 2-2 piezoelectric composite material is selected to prepare the trilaminar vibrator according to the results of finite element simulation. Three groups of the trilaminar vibrators are prepared in this experiment to ensure the accuracy of the experiment. Each group has two samples of different materials, including a 2-2 piezoelectric composite trilaminar vibrator and a piezoelectric ceramic trilaminar vibrator, and the total number of samples is six. The 2-2 piezoelectric composite trilaminar vibrator is used to study the bending vibration characteristics of the novel piezoelectric composite vibrator, while the piezoelectric ceramic trilaminar vibrator is used for comparative experiments.

**Figure 8:**
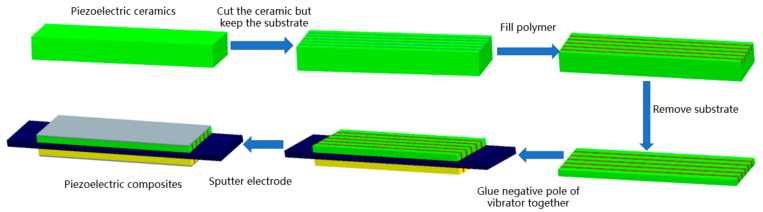
Flow chart of 2-2 piezoelectric composite trilaminar vibrator.

The 2-2 piezoelectric composite trilaminar vibrator comprised a piezoelectric phase, a polymer phase, and surface electrodes with the following structural parameters: length = 50 mm, width = 14.8 mm, height = 2 mm, column width = 2 mm and joint-cutting width = 0.56 mm. The PZT-5A piezoelectric ceramics, silicone rubber and silver paste are selected as the piezoelectric phase, polymer phase and surface electrode, respectively. The 2-2 piezoelectric composites are prepared by the “dice-filled” method [25,26,27] and the preparation process is shown in Figure 8.

The detailed preparation process is as follows:

(1) A cutting machine is used to cut the piezoelectric ceramic to form a piezoelectric ceramic column, but the substrate is retained.
(2) The silicone rubber is poured between the piezoelectric ceramic columns.
(3) The substrate is removed, but the original negative electrode is retained.
(4) The negative electrodes of the two single-chip vibrators are bonded to the metal sheet to form a trilaminar vibrator, and then the other two electrodes are sputtered.

**Figure 9:**
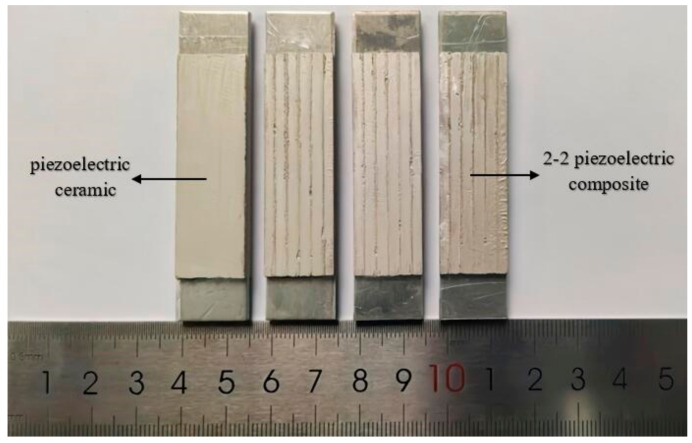
The fabricated trilaminar vibrator samples.

The 2-2 piezoelectric composite trilaminar vibrator samples are fabricated in accordance with the above preparation method as shown in Figure 9. Then, the bending vibration characteristics of the vibrator are tested by using an impedance analyzer and a laser vibrometer under the fixed boundary condition.

**Figure 10:**
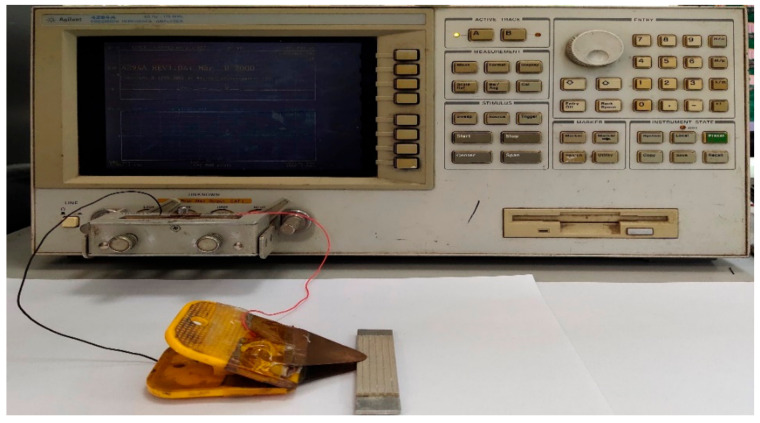
The Aligent 4294 impedance analyzer.

Three groups of 2-2 piezoelectric composite and piezoelectric ceramic trilaminar vibrators are tested using an impedance analyzer (Agilent 4294A, Agilent Technologies, Inc., Santa Clara, CA, USA). The trilaminar vibrator is connected to the impedance analyzer according to the connection mode shown in Figure 10, and the high voltage terminal H and low voltage terminal L are connected to the upper and lower surfaces of the trilaminar vibrator, respectively. Such an impedance analyzer adopts the technology of automatic balance bridge and measures based on Ohm’s law. The impedance, bandwidth and electromechanical coupling coefficient of the vibrator are calculated by adding an AC source on the surface of the trilaminar vibrator. These parameters can be automatically read in the computer through the external display. The resonant frequency and bandwidth of the vibrators are directly measured. Meanwhile, the effective electromechanical coupling coefficients are calculated using Equation (1), where *f_s_* and *f_p_* refer to the resonant frequency and anti-resonant frequency, respectively. The test results are shown in Table 2 and Table 3. The table demonstrates that the test results of the three groups of vibrators are basically consistent.

(1) $$k_{e}=\sqrt{1-\frac{f_{s}{}^{2}}{f_{p}{}^{2}}}$$

**Figure 11:**
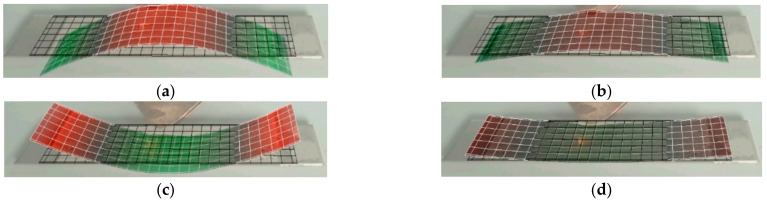
The vibration mode diagram of the trilaminar vibrator: (**a**) 1/4 period; (**b**) 2/4 period; (**c**) 3/4 period; (**d**) 4/4 period.

The vibration modes and displacements of three groups of vibrators are observed with a laser vibrometer (PSV-400, Polytec, Inc., Karlsruhe, Germany). The vibration mode of one period of vibrator is shown in Figure 11. The figure shows that the trilaminar vibrator has a pure bending vibration mode in the whole period and can produce large vibration displacement. The maximum vibration displacement of the vibrator is read, as shown in Table 2 and Table 3 above.

**Figure 12:**
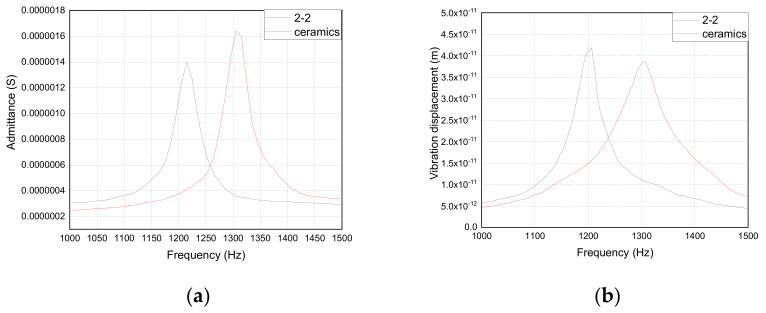
Test results of admittance curve and vibration displacement curve: (**a**) admittance curve; (**b**) vibration displacement curve.

The average piezoelectric parameters of the 2-2 and ceramic vibrators are shown in Table 4. The admittance and vibration displacement curves of the 2-2 piezoelectric composite and piezoelectric ceramic trilaminar vibrators obtained by experiments are shown in Figure 12a,b, respectively. Table 4 and Figure 12 show that the 2-2 piezoelectric composite trilaminar vibrator has lower resonance frequency and larger vibration displacement compared with that of the piezoelectric ceramic vibrator, and its bandwidth and electromechanical coupling coefficient are improved to a certain extent.

Comparing Figure 7 with Figure 12, it can be seen that the experimental results are basically consistent with the simulation results. Some errors may occur because the simulation results are too ideal. The two main errors are as follows: (1) the experimental parameters are not consistent with the simulation parameters; (2) the fixture used to fix the boundary is not ideal.

## 5. Discussion

The 2-2 piezoelectric composites have the advantages of both the piezoelectric ceramics and the polymeric materials which not only have low density, but have a simple manufacturing process. The 2-2 piezoelectric composites mainly have the following advantages compared with the piezoelectric ceramics [28]. Firstly, the 2-2 piezoelectric composites have lower P-wave velocity compared with the piezoelectric ceramics, and material with lower P-wave velocity can obtain lower resonant frequency. Therefore, the resonant frequency of the 2-2 piezoelectric composites is lower than that of the piezoelectric ceramics. Secondly, the Q value of 2-2 piezoelectric composites is much lower than that of the traditional piezoelectric ceramics which makes the 2-2 piezoelectric composites have higher bandwidth. Thirdly, the 2-2 piezoelectric composite has a smaller plane electromechanical coupling coefficient compared with the piezoelectric ceramics and its energy is more concentrated in the thickness mode, so it has a higher thickness electromechanical coupling coefficient. Finally, the stiffness of 2-2 piezoelectric composites is smaller compared with that of the piezoelectric ceramics; thus, the 2-2 piezoelectric composites can produce larger bending deformation under the same voltage, and then can produce more significant vibration displacement.

## 6. Conclusions

In this work, the 2-2 piezoelectric composite trilaminar vibrator is designed and fabricated through the combination of the finite element simulation and experiment. Firstly, the bending vibration characteristics of the trilaminar vibrator are simulated and analyzed. The results show that the trilaminar vibrator has lower resonance frequency and larger vibration displacement under the fixed boundary condition compared with that of the free boundary condition. Then, the 2-2 piezoelectric composite and the piezoelectric ceramic trilaminar vibrators are fabricated and their bending vibration characteristics are tested under the fixed boundary condition. The experimental results show that the 2-2 piezoelectric composite vibrator has lower resonance frequency, higher electromechanical coupling coefficient and larger vibration displacement compared with the piezoelectric ceramic vibrator. Therefore, the 2-2 piezoelectric composite trilaminar vibrator has more excellent performance, which is a good choice for making a low-frequency transducer.

## Figures and Tables

**Table 1:** Material parameters.

Parameters	PZT-5A	Parameters	Silicon Rubber	Aluminum
*ρ* (kg/m^3^)	7750	*ρ* (kg/m^3^)	1000	2790
$c_{11}^{E}$ (10^10^ N/m^2^)	12.1	*EX* (10^10^ N/m^2^)	2.55 × 10^−^^4^	7.15
$c_{12}^{E}$ (10^10^ N/m^2^)	7.54	*σ*	0.48	0.34
$c_{13}^{E}$ (10^10^ N/m^2^)	7.52	$c_{11}^{E}$ (10^10^ N/m^2^)	0.004	
$c_{33}^{E}$ (10^10^ N/m^2^)	11.1	$c_{12}^{E}$ (10^10^ N/m^2^)	0.0023	
$s_{11}^{E}$ (10^−12^ m^2^/N)	16.4	$s_{11}^{E}$ (10^−12^ m^2^/N)	4 × 10^5^	
$s_{12}^{E}$ (10^−12^ m^2^/N)	−5.74	$s_{12}^{E}$ (10^−12^ m^2^/N)	2.3 × 10^5^	
$s_{13}^{E}$ (10^−12^ m^2^/N)	−7.22	$\varepsilon_{33}^{S}$/*ε*_0_	3.3	
$s_{33}^{E}$ (10^−12^ m^2^/N)	18.8	$\varepsilon_{33}^{T}$/*ε*_0_	3.3	
*d*_31_ (10^−12^ C/N)	470			
*d*_33_ (10^−12^ C/N)	−171			
*e*_31_(C/m^2^)	−5.4			
*e*_33_(C/m^2^)	15.8			
$\varepsilon_{33}^{S}$/*ε*_0_	830			
$\varepsilon_{33}^{T}$/*ε*_0_	1700			

**Table 2:** Performance test results of 2-2 piezoelectric composite vibrators.

Number	Resonant Frequency (Hz)	Anti-Resonant Frequency (Hz)	Bandwidth (Hz)	Electromechanical Coupling Coefficient	Maximum Vibration Displacement (m)
1	1209	1261	122.5	0.284	4.21 × 10^−11^
2	1195	1245	118.3	0.280	4.15 × 10^−11^
3	1205	1256	121.6	0.282	4.18 × 10^−11^

**Table 3:** Performance test results of piezoelectric ceramics vibrators.

Number	Resonant Frequency (Hz)	Anti-Resonant Frequency (Hz)	Bandwidth (Hz)	Electromechanical Coupling Coefficient	Maximum Vibration Displacement (m)
1	1288	1327	82.3	0.241	3.89 × 10^−11^
2	1282	1322	79.6	0.244	3.84 × 10^−11^
3	1285	1324	80.3	0.241	3.85 × 10^−11^

**Table 4:** The average piezoelectric parameters of the 2-2 and ceramic vibrators.

Type	Resonant Frequency (Hz)	Anti-Resonant Frequency (Hz)	Bandwidth (Hz)	Electromechanical Coupling Coefficient	Maximum Vibration Displacement (m)
2-2	1203	1254	120.8	0.282	4.18 × 10^−11^
ceramic	1285	1324	80.7	0.242	3.86 × 10^−11^

## Data Availability

The data presented in this study are available on request from the corresponding author.
